# Reassurance or Redundancy: A Retrospective Observational Study of the Clinical Utility of Abdominal Radiographs in an Australian Emergency Department

**DOI:** 10.7759/cureus.117949

**Published:** 2026-10-15

**Authors:** Aditya D Rao, Julian Williams

**Affiliations:** 1 Emergency Department, Royal Brisbane and Women's Hospital, Brisbane, AUS

**Keywords:** abdomen, abdominal radiography, compliance, computed tomography, emergency department

## Abstract

Introduction

Acute abdominal pain accounts for a significant proportion of emergency department (ED) presentations, and abdominal radiographs or X-rays (AXR) remain among the most frequently performed investigations for this presentation. Although radiographs carry a lower radiation dose than computed tomography (CT), this is offset by comparatively poor diagnostic yield, and CT has been shown to improve diagnostic accuracy and clinical outcomes in acute abdominal pain. The Royal College of Radiologists (RCR) iRefer guidelines define appropriate indications for abdominal radiographs to maximise its clinical utility and are endorsed for use in Australia by the Royal Australian and New Zealand College of Radiologists. This study aimed to assess whether abdominal radiograph requests at an Australian tertiary ED met RCR iRefer indications and whether radiograph findings informed management or led to further imaging within the same presentation.

Methods

This retrospective study reviewed all abdominal radiographs performed at the Royal Brisbane and Women's Hospital ED between 1 January and 30 June 2025, identified via the Radiology Information System. Adult patients (≥18 years) imaged for acute abdominal pain were included; those imaged to monitor known pathology were excluded. Request-form indications were compared against RCR iRefer guidelines. Clinical notes were reviewed to determine whether radiograph findings directly informed management or whether further imaging was required.

Results

Of 191 radiographs identified, 162 met inclusion criteria. The mean age was 48.3 years (range 18-99), with an approximately equal sex distribution. Overall, 85.18% (n=138) of requests were iRefer-compliant, with non-compliance (14.82%, n=24) largely attributable to inpatient-initiated and device-position check requests. The leading indications were exclusion of constipation (37.03%) and suspected bowel obstruction or volvulus (29.6%). No acute pathology was identified in 45.06% (n=73) of studies. Constipation and faecal loading were seen in 36.41% (n=59) of studies and bowel obstruction in 7.4% (n=12). Radiograph findings directly informed management in 75.9% (n=123) of cases (79 discharged, 44 admitted). Further imaging was required in 24.10% (n=39): 34 underwent CT, four underwent ultrasound, and one obtained both modalities. This further imaging identified a positive finding in 26 patients (66.6%), while 13 (33.3%) had no defined aetiology. Notably, clinically significant pathologies, including bowel obstruction, new inflammatory bowel disease, and previously undetected foreign bodies, were identified on further imaging in a subset of patients despite an initially negative or non-specific radiograph.

Conclusion

Abdominal radiography in this centre was largely compliant with the iRefer guidelines, but diagnostic yield was limited, and a meaningful proportion of patients required subsequent cross-sectional imaging, some of which revealed pathology not apparent on the initial radiographs. These findings support closer adherence to core iRefer indications and consideration of more selective first-line abdominal radiograph use, with earlier consideration of CT in patients with a high pre-test probability of significant pathology.

## Introduction

Acute abdominal pain accounts for 5-10% of emergency department (ED) presentations, and rapid, accurate diagnosis is essential to guide immediate management and support optimal patient outcomes [1]. Abdominal X-rays (AXR) or radiographs remain among the most frequently performed radiological investigations for this presentation. Originally used to identify foreign bodies and bony injuries, their role has since expanded to include the diagnosis of renal calculi, intestinal obstruction, volvulus, colitis, and free intraperitoneal air indicating perforation [2].

More recently, however, the diagnostic utility of AXR has come under scrutiny. While its radiation dose of 0.7 millisieverts (mSv) is lower than that of an abdominal computed tomography (CT) scan at 10 mSv, this advantage is increasingly offset by growing evidence of its comparatively poor diagnostic yield [3]. By contrast, abdominal CT scans have been shown to improve diagnostic accuracy, reduce length of hospital stay, and lower mortality in patients presenting with acute abdominal pain [4].

Given this combination of limited diagnostic yield and the frequent need for subsequent CT imaging, the Royal College of Radiologists (RCR) developed the iRefer guidelines to define appropriate indications for AXR and maximise its clinical utility. Australia does not currently maintain an independent set of guidelines for abdominal radiograph use. Instead, the Royal Australian and New Zealand College of Radiologists (RANZCR) has endorsed the adoption of the RCR guidelines and continues to oversee their ongoing alignment with current, evidence-based practice [5].

While a small number of Australian studies have been performed in this area, published data on AXR appropriateness and downstream imaging in the Australian emergency setting remain limited [6].

The primary aim of this study was to review abdominal radiographs ordered from the Royal Brisbane and Women’s Hospital (RBWH) ED over a six-month period to identify the indications for imaging and assess their appropriateness against the RCR iRefer guidelines. The secondary aim was to determine whether radiograph findings were documented as informing patient management and whether further imaging was required within the same presentation.

## Materials and methods

This retrospective study reviewed all abdominal radiographs performed at the RBWH ED between 1st January and 30th June 2025, identified through the Radiology Information System (RIS) Karisma.

Adult patients (≥18 years) who underwent an abdominal radiograph for an ED presentation of acute abdominal pain were included. Patients under 18 years of age and those imaged to monitor known pathology (e.g., renal calculi) were excluded.

Electronic request forms were reviewed to identify the documented indication for each radiograph, which were then compared against the RCR iRefer guidelines to assess the appropriateness of each request [5]. Request indications were assessed by a single reviewer, with no independent second review.

Clinical notes from the corresponding presentation were reviewed via the Emergency Department Information System (EDIS) to determine whether radiograph findings directly informed patient management and whether further imaging, most commonly CT, was required within the same presentation. Three outcomes were assessed separately: appropriateness (concordance of the documented request indication with iRefer guidelines); diagnostic yield (whether the radiograph showed an acute abnormality); and impact on management or patient disposition. A radiograph was considered to have directly informed management if its result contributed to the decision to admit or discharge without further investigations. This was determined retrospectively from clinical documentation, not against a predefined utility measure. Where CT was performed, we further assessed whether this was undertaken to confirm a suspected finding on the radiograph or to investigate persistent symptoms in the absence of a radiographic explanation.

## Results

Of 191 abdominal radiographs identified, 162 met inclusion criteria for the study. Participant age ranged from 18 to 99 years (mean 48.3 years), with an approximately equal sex distribution (51% male, 49% female). Overall, 85.18% (n=138) of requests were compliant with iRefer guidelines (Table 1).

**Table 1:** Key indications for plain abdominal imaging from Royal College of Radiologists iRefer guidelines

No.	Indication
1	Clinical suspicion of obstruction
2	Acute exacerbation of inflammatory bowel disease
3	Palpable mass (specific circumstances)
4	Constipation (specific circumstances)
5	Acute and chronic pancreatitis (specific circumstances)
6	Sharp/poisonous foreign body
7	Smooth and small foreign body (e.g., coin, battery) (specific circumstances)
8	Blunt or stab abdominal injury (specific circumstances)

The remaining 14.82% (n=24) were non-compliant, primarily reflecting requests initiated by inpatient teams or for device-position checks. The most common indications were exclusion of constipation (37.03%) and suspected bowel obstruction or volvulus (29.6%). Foreign body exclusion or localisation accounted for 8.02% (n=13) of requests, with patients suspected of having swallowed foreign bodies obtaining a chest radiograph in addition. A further 11.11% (n=18) were requested to assess for toxic megacolon in the context of an inflammatory bowel disease exacerbation, and 9.25% (n=15) were performed to confirm device position, including percutaneous endoscopic gastrostomy (PEG) or jejunostomy (PEJ) tubes, Tenckhoff catheters, and ureteric stents. One radiograph was requested specifically to assess for contrast extravasation from a ureteric stent in a patient with postoperative pain within 24 hours of insertion (Table 2).

**Table 2:** Indications for abdominal radiograph requests and compliance with Royal College of Radiologists iRefer guidelines IBD: inflammatory bowel disease

Indication	% of requests (n)	iRefer compliant
Constipation exclusion	37.03% (60)	Yes
Bowel obstruction/volvulus	29.6% (48)	Yes
Toxic megacolon (IBD exacerbation)	11.11% (18)	Yes
Device-position check	9.25% (15)	No
Foreign body exclusion	8.02% (13)	Yes
Other (inpatient-initiated request)	4.99% (8)	No
Total	100% (162)	85.18% compliant

A total of 45.06% (n=73) of studies showed no acute pathologies. Radiographic findings were consistent with constipation or faecal loading in 36.41% (n=59) of cases, all of which were managed conservatively. Bowel obstructions were identified in 7.4% (n=12) of cases (Figure 1).

**Figure 1:**
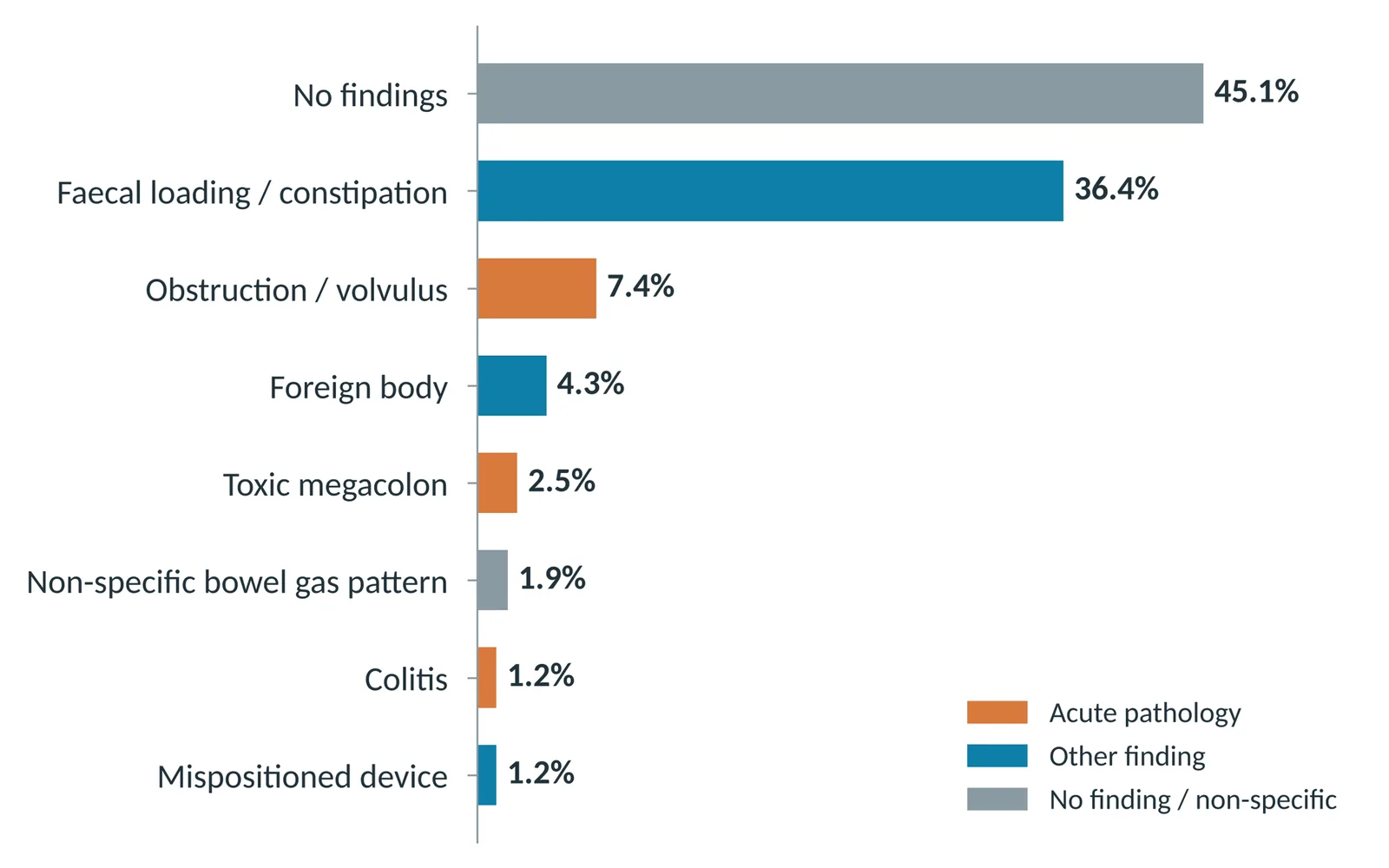
Findings on initial abdominal radiographs shown as a percentage of all radiographs included. Orange bars indicate acute pathology (bowel obstruction/volvulus, toxic megacolon and colitis), teal bars indicate other findings, and grey bars indicate no finding or a non-specific bowel gas pattern.

Radiograph findings directly informed patient management in 75.9% (n=123) of cases, resulting in discharge with safety-netting advice for 79 patients and inpatient admission for 44. Further imaging was required in 24.10% (n=39) of cases, either to confirm a positive radiographic finding or to investigate persistent symptoms despite a negative or non-specific study. Of these 39 patients, 34 underwent CT, four underwent ultrasound, and one obtained both modalities. Further imaging identified a positive finding in 26 patients (66.6%), while 13 (33.34%) had no clearly defined aetiology for their abdominal pain.

Within this subgroup requiring further imaging, five categories were identified based on the initial radiograph finding. First, 17 patients (43.58%) underwent further imaging despite a negative radiograph due to persistent symptoms. While most subsequent studies were negative or showed features of colitis, positive findings included a bowel obstruction (n=1), new inflammatory bowel disease (n=2), and a previously undetected foreign body (n=1). Second, nine patients (23.07%) with radiographs suggestive of constipation underwent further imaging due to disproportionate pain, revealing a closed-loop bowel obstruction (n=1) and an intra-abdominal collection (n=1), both requiring surgical management. Third, of seven patients (17.94%) who underwent CT for a radiograph suspicious for bowel obstruction, the diagnosis was confirmed in four and colitis was identified in the remaining three. Fourth, in two (5.13%) patients imaged further to localise a foreign body, CT identified a secondary foreign body proximal to the one seen on the initial radiograph in both cases. Finally, four patients (10.26%) underwent further imaging for non-specific radiographic findings, such as abnormal bowel gas patterns, none of which had positive findings on the subsequent CT scans (Figure 2).

**Figure 2:**
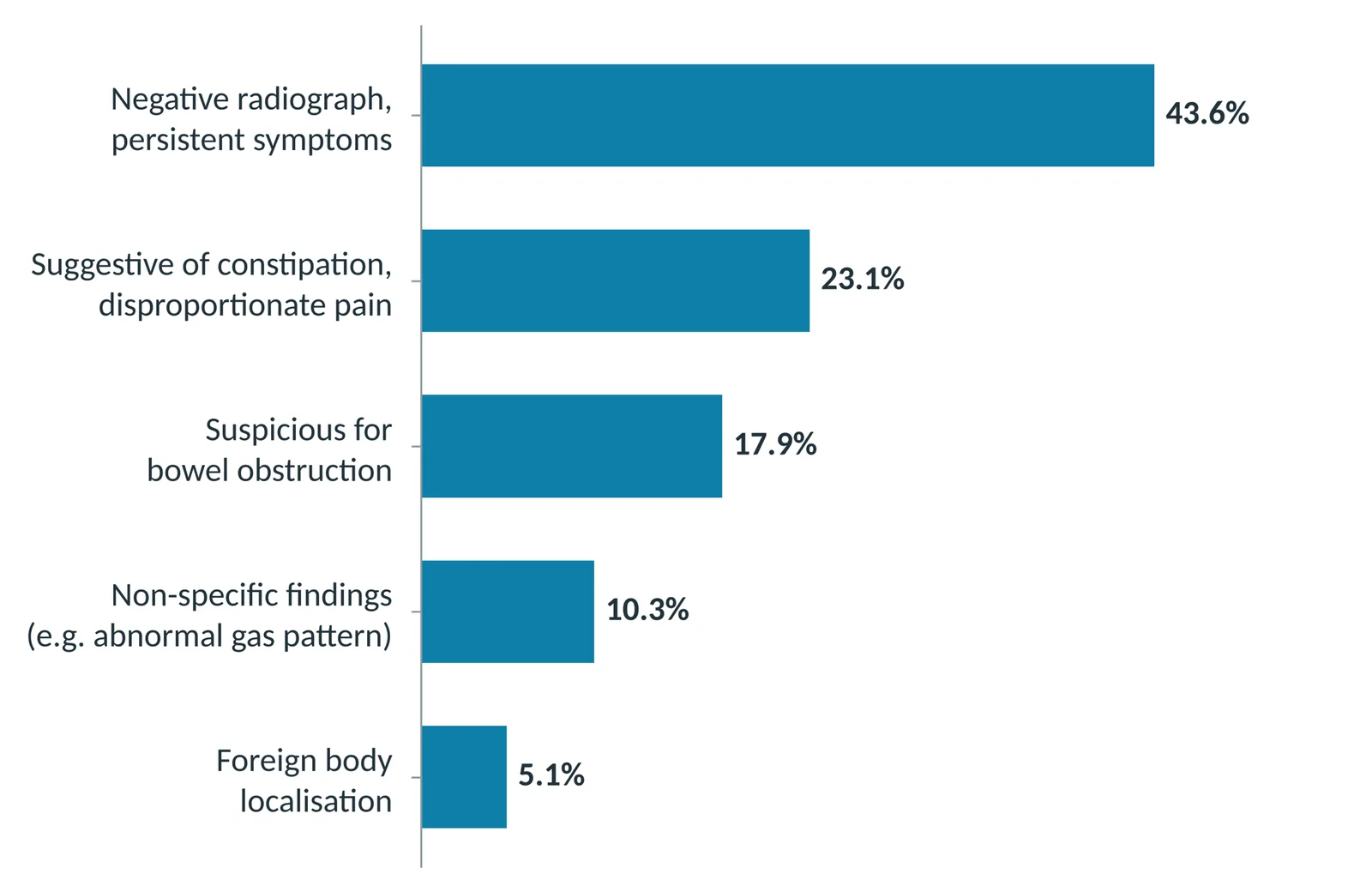
Patients requiring further imaging within the same presentation, grouped by the initial radiograph finding and shown as a percentage of total patients (n=39).

## Discussion

This six-month retrospective study of 162 abdominal radiographs performed in the RBWH ED found that the large majority of imaging requests (85.18%) were compliant with the RCR iRefer guidelines. Results also showed that just under half of studies (45.06%) were negative, that AXR findings directly influenced management in over three-quarters of patients and that approximately one in four patients required further imaging within the same presentation. These findings are broadly consistent with, but also usefully diverge from, the existing literature assessing abdominal radiograph use against iRefer criteria and raise several points relevant to local practice.

Guideline adherence

Our compliance rate of 85.18% sits within the range reported by comparable single-centre studies internationally. A United Kingdom (UK) based audit from Gateshead reported 88% iRefer compliance, with intestinal obstruction and perforation as the leading indications, closely resembling our own indication profile [7]. A larger Welsh study with a sample size of 242 participants reported 89.67% compliance to guidelines, with intestinal obstruction again being the predominant indication, and a Scottish tertiary hospital found a compliance rate of 92.9%, with the leading indications being bowel perforation/obstruction, renal colic, foreign body and constipation [1,8].

Non-compliant requests in our cohort were largely attributable to inpatient team-initiated requests and device-positioning checks. These indications fall outside the core iRefer criteria but nonetheless represent widely used, low-risk clinical practice for confirming device placement in the emergency and inpatient settings. This suggests that a proportion of our ‘non-compliant’ studies reflect a limitation of applying an emergency-focused guideline to a mixed cohort of both emergency and referred inpatients. Future local audit cycles may benefit from separately categorising device checks and inpatient-initiated requests, rather than scoring them against the iRefer guidelines designed for de novo emergency presentations of abdominal pain.

In circumstances where genuine stray from guideline adherence does occur, literature shows that targeted intervention has proven effective. A UK-based quality improvement initiative reduced inappropriate AXR requests in a surgical admission unit from 69% to 11% through locally agreed-upon guidelines, education, posters, and changes to the electronic request form [9]. Similarly, a London district general hospital audit found that clinician education and poster-based guideline dissemination increased iRefer-compliant requesting and the subsequent diagnostic yield [10].

Diagnostic yield and clinical utility

Nearly half of the radiographs in our cohort (45.06%) showed no positive findings, and where positive findings were present, they were dominated by constipation/faecal loading (36.41%) rather than by more clinically urgent pathology such as bowel obstructions [11]. This pattern is consistent with a substantial body of literature documenting the limited diagnostic yield of radiographs in acute abdominal pain.

Studies on diagnostic accuracy directly comparing AXR with CT imaging have consistently found a markedly lower sensitivity in abdominal radiographs. One prospective comparison evaluates diagnostic accuracy between the modalities and found a sensitivity of 46% for radiographs and 75% for CT imaging, both with similar specificity of 87%. A systematic review of comparable studies found AXR sensitivity as low as 30%, against CT sensitivity of up to 96% [12]. A diagnostic accuracy study from the Netherlands similarly demonstrated that CT-based strategies outperformed isolated radiograph-based pathways in identifying emergent abdominal pathologies, and that a conditional strategy of CT imaging only after a negative ultrasonography study achieved the best balance of sensitivity and minimising radiation exposure [13].

It is also important to note that the interpretation of faecal loading on AXR has itself been shown to carry considerable inter-observer variability and poor correlation with colonic transit time, another factor putting into question the reliability of this finding as a basis for clinical decision-making [14].

Impact on management and subsequent imaging

AXR findings were reported as directly affecting disposition (discharge with safety-netting or admission) in 75.9% of our cohort. This figure should be interpreted cautiously, as it reflects a documented association between radiograph result and patient disposition rather than a measure of diagnostic utility.

The requirement for further imaging in 24.10% of our cohort is consistent with comparable studies such as that by Salman et al., which have reported rates of subsequent imaging around 29% to obtain a definitive diagnosis [7]. This reflects the well-recognised issue that abdominal radiographs frequently require confirmatory or additional cross-sectional imaging, given its established low diagnostic yield, which causes a subsequent downstream imaging burden in the emergency setting. It is important to note that a meaningful minority of patients in our study that obtained further imaging despite negative radiographs were found to have clinically significant pathologies, including bowel obstructions, new inflammatory bowel disease and, in two cases, a secondary foreign body not visualised on the original film. This is consistent with the broader evidence base indicating that AXR carries a clinically important false-negative rate and reiterates that a normal or indeterminate AXR should not be used to exclude significant pathology in a patient with persisting symptoms [12].

Radiation and resource implications

Two-step imaging pathways involving both abdominal radiographs and CT imaging carry cumulative radiation, departmental resource utilisation, and clinician time costs [15]. Given that a substantial proportion of our cohort proceeded to CT due to ongoing clinical concerns, and that further imaging identified a positive finding in two-thirds of these selected patients, our data raise the possibility that more selective first-line radiograph use may be appropriate where pre-test probability of CT-detectable pathology is high.

Limitations

This study has limitations secondary to its retrospective, single-centre design with a limited sample size. iRefer compliance was determined by reviewing documentation from request forms, which may not fully capture the requesting clinician's reasoning and is subject to documentation bias. Request indications and clinical records were reviewed by a single reviewer without independent verification, which may introduce observer bias. Imaging findings and their impact on management were also determined by retrospective chart review rather than a prospective clinician-reported outcome, which makes it challenging to determine the definitive causal contribution to the management decision. Additionally, the impact on management was inferred from the absence of further imaging and the recorded disposition and therefore includes negative radiographs that supported discharge, which may overestimate the clinical utility of abdominal radiography.

## Conclusions

This retrospective study found that abdominal radiography in our centre was largely compliant with the RCR iRefer guidelines. However, diagnostic yield was limited, with almost half of studies showing no acute pathology and nearly a quarter of patients requiring further imaging within the same presentation. Returning to our opening question, abdominal radiography was neither uniformly reassuring nor redundant. In most patients, no further imaging was required, although our study cannot show that the radiograph itself provided the reassurance. Among those who underwent further imaging, the second test often added nothing the radiograph had not already shown and so duplicated its information, but in a substantial minority, it identified new, clinically significant pathology. Consistent with international evidence, this study demonstrates that while abdominal radiograph findings directly informed management in the majority of cases, a high rate of negative or non-specific results created a downstream requirement for subsequent imaging, in some cases resulting in delayed identification of significant pathology. These findings support continued local education around iRefer indications, particularly clarifying expectations for inpatient-initiated and device-positioning check requests.

Additionally, consideration of more direct escalation to CT imaging in patients with a high pre-test probability of significant pathology could significantly reduce cumulative radiation exposure and diagnostic delay. Future research incorporating prospective clinician-reported outcomes and multi-centre data would help clarify the true impact of abdominal radiography on ED management pathways and confirm whether these findings can be generalised beyond a single tertiary centre.

## References

[1] Hassan T, Kumwenda M, Qutab S, Naqvi SZ, Rashid H, Johnson S (2022). Are we following iRefer guidelines from the Royal College of Radiology when requesting abdominal X-rays?. Cureus.

[2] James B, Kelly B (2013). The abdominal radiograph. Ulster Med J.

[3] Laméris W, van Randen A, van Es HW (2009). Imaging strategies for detection of urgent conditions in patients with acute abdominal pain: diagnostic accuracy study. BMJ.

[4] Ng CS, Watson CJ, Palmer CR (2002). Evaluation of early abdominopelvic computed tomography in patients with acute abdominal pain of unknown cause: prospective randomised study. BMJ.

[5] Reid C, Rafique A, Fang C (2016). Indications for Plain Abdominal Films From the Emergency Department. The Royal College of Radiologists.

[6] Bunting C, Smith E, Dunham F, Simpson S, Truter P (2023). Adult patients with constipation presenting to the emergency department: can care be improved?. Emerg Med Australas.

[7] Salman R, Musa A, Abdelkarim M, Dennison C (2018). An audit of indications for plain abdominal films from the emergency department in Queen Elizabeth Hospital-Gateshead during May 2017. EPOS.

[8] Nolan O, Anderson R, Gall L, Khan K (2022). Abdominal X-rays in emergency department: assessing compliance with RCR iRefer guidelines in a tertiary hospital’s emergency department. Br J Surg.

[9] Barrington M, Ragatha P, O'Malley EJ, Bethune R (2025). Reducing inappropriate abdominal X-ray requests in a surgical admissions unit: a quality improvement project. Cureus.

[10] Kyriakides J, Khamar R, Khani A, Khatkar H (2022). A quality improvement project: reducing the number of unnecessary plain abdominal radiographs performed in the emergency department of a London district general hospital. J Family Med Prim Care.

[11] Frager D, Medwid SW, Baer JW, Mollinelli B, Friedman M (1994). CT of small-bowel obstruction: value in establishing the diagnosis and determining the degree and cause. AJR Am J Roentgenol.

[12] Alshamari M, Norrman E, Geijer M, Jansson K, Geijer H (2016). Diagnostic accuracy of low-dose CT compared with abdominal radiography in non-traumatic acute abdominal pain: prospective study and systematic review. Eur Radiol.

[13] Masoodi S, PTM Van, Farzaneh SB, Cribb E, Pawa N (2022). An audit of indications for emergency abdominal X-rays. BJS.

[14] Cowlam S, Vinayagam R, Khan U (2008). Blinded comparison of faecal loading on plain radiography versus radio-opaque marker transit studies in the assessment of constipation. Clin Radiol.

[15] Brenner DJ, Hall EJ (2007). Computed tomography—an increasing source of radiation exposure. N Engl J Med.

